# Dose–Volume Constraints for Thoracic, Abdominal, and Pelvic Carbon Ion Radiotherapy: A Literature Review

**DOI:** 10.1002/cam4.70840

**Published:** 2025-03-28

**Authors:** Maria Varnava, Mutsumi Tashiro, Masahiko Okamoto, Ken Ando, Nobutero Kubo, Hidemasa Kawamura, Masahiro Onishi, Kei Shibuya, Takuya Kumazawa, Takeru Ohtaka, Tatsuya Ohno

**Affiliations:** ^1^ Gunma University Heavy Ion Medical Center Maebashi Gunma Japan; ^2^ Department of Radiation Oncology Gunma University Graduate School of Medicine Maebashi Japan

**Keywords:** abdominal cancer, carbon ion radiotherapy, dose–volume constraints, organs at risk, pelvic cancer, thoracic cancer

## Abstract

**Background:**

Applying dose–volume constraints is extremely important in ensuring the safe use of radiotherapy. However, constraints for carbon ion radiotherapy (CIRT) have not been established yet. This review aims to summarize dose–volume constraints for thoracic, abdominal, and pelvic CIRT that have been identified through previous research based on the Japanese models for relative biological effectiveness (RBE).

**Results:**

Constraints are reported for the lungs, liver, stomach, gastrointestinal tract, rectum, sigmoid, bladder, nerves, rib, femoral head, sacrum, and skin. The constraints are classified into hard and soft to aid in determining whether priority should be given to the target coverage or organ‐at‐risk (OAR) sparing during treatment planning.

**Conclusions:**

Further research is necessary to verify the applicability of the reported constraints and to identify constraints for the OARs that have not been investigated yet.

## 1 Introduction

Carbon ion radiotherapy (CIRT) has been slowly gaining ground in radiation therapy. Currently, there are 15 CIRT institutions in operation worldwide, with 7 of them located in Japan [1]. The first CIRT institution started operating in 1994 in Japan, making Japan the country with the longest clinical experience in CIRT, followed by Germany.

Compared with X‐ray radiotherapy (XRT) and proton therapy, CIRT exhibits a higher linear energy transfer, resulting in higher biological effectiveness [2]. Promising outcomes have already been shown for the treatment of radioresistant tumors [3]. Moreover, carbon ions exhibit a characteristic Bragg peak that leads to low doses delivered to normal tissues proximal to the target and high doses delivered to the target [2]. The way dose is distributed to the organs at risk (OARs) and target depends on the distance between them and the beam delivery technique. Consequently, these influence the degree of OAR morbidities and target control. Therefore, the use of dose–volume constraints is important in optimizing treatment plans.

Contrary to XRT, there are no established dose–volume constraints for CIRT. In XRT, dose–volume histogram (DVH) parameters associated with the development of adverse effects have been suggested based on the empirical clinical practice of radiotherapy institutions and long‐term clinical toxicity data [4, 5]. Unfortunately, because of the biological and qualitative differences between XRT and CIRT, the same constraints in XRT cannot be applied to CIRT.

The use of relative biological effectiveness (RBE)‐weighted doses is recommended for use in particle therapy by the International Commission on Radiation Units and Measurements and clinical practice [6]. The radiobiological properties of carbon ions are described using complex RBE models. Currently, three RBE models are used in clinical practice: two Japanese models, the mixed‐beam model and the modified microdosimetric kinetic model, and the local effect model (LEM), which is mainly applied in European and Chinese institutions [7, 8, 9, 10]. The Japanese models and LEM differ regarding their physical and mathematical assumptions and consider different endpoints [11]. Therefore, the RBE‐weighted dose depends on the RBE model adopted at each institution. This indicates that Japanese‐based constraints are not equivalent to LEM‐based constraints, and separate constraints should be established for the Japanese models and LEM.

Previous articles have summarized Japanese and LEM‐based constraints for the head‐and‐neck cancer CIRT [11, 12, 13]. The purpose of this review is to present the dose–volume constraints reported for thoracic, abdominal, and pelvic CIRT that were determined based on the Japanese RBE models [14, 15, 16, 17, 18, 19, 20, 21, 22, 23, 24, 25, 26, 27, 28, 29, 30, 31, 32, 33, 34, 35]. We also report any lacking information necessary to provide a thorough list of constraints for all related OARs. The list of constraints can be used as a supplementary guide during treatment planning.

The following sections summarize the literature on dose–volume constraints for OARs in thoracic, abdominal, and pelvic CIRT. The CIRT dose is expressed in units of Gy (RBE), defined as the product of the physical dose and carbon ion RBE. The RBE is evaluated based on the biological NIRS (National Institute of Radiological Sciences, Japan) model, which uses the linear‐quadratic (LQ) model [36].

## 2 Lungs

Radiation‐induced lung injury is a common adverse effect in radiotherapy. It can manifest as an acute complication, called radiation‐induced pneumonitis (RP), and/or a late complication, called radiation‐induced fibrosis [37, 38]. Both are serious and life‐threatening, and fibrosis could lead to a compromised physiological function of the lung tissue [38].

A study investigated prognostic factors associated with RP for 65 patients with locally advanced non‐small‐cell lung cancer (NSCLC), including patients with lymph node metastasis [14]. The patients were treated using 68–76 Gy (RBE) in 16 fractions. Symptomatic radiation pneumonitis was graded according to the Common Terminology Criteria for Adverse Effects (CTCAE). Receiver operating characteristic (ROC) analysis revealed cut‐off values for grade ≥ 2 RP for various DVH parameters: mean dose (Dmean) ≥ 12.5 Gy (RBE), volume receiving 5 Gy (RBE) (V5) ≥ 28.8%, V10 ≥ 29.9%, V20 ≥ 20.1%, and V30 ≥ 15.0%. Multivariate analysis showed V30 to be an independent risk factor. The incidences of grade ≥ 2 RP for V30 ≥ 15.0% and V30 < 15.0% were 28.0% and 0%, respectively.

Another study investigated predictive factors for RP [15]. Data of 98 patients with solitary lung tumors and no lymph node metastasis were analyzed. The prescription dose was 50 Gy (RBE) delivered in a single fraction. RP was evaluated based on the CTCAE. Univariate analysis showed that the lung V5–30 and mean lung dose (MLD) were significantly higher in patients with grade ≥ 2 RP than in patients with grade 0–1 RP. ROC analysis revealed cut‐off values for grade ≥ 2 RP: V5 > 11%, V10 > 9.4%, V15 > 7.8%, V20 > 6.8%, V25 > 4.5%, V30 > 3.5%, and MLD > 3 Gy (RBE). None of the patients with DVH parameters less than their corresponding cut‐off value developed grade ≥ 2 RP.

Two studies reported lung indices related to the severity of radiation‐induced lung injury [16, 17]. Nakajima et al. evaluated RP grade progression in 29 patients with NSCLC and interstitial lung disease, including patients with lymph node metastasis [16]. The patients were treated using 52.8–72.6 Gy (RBE) in 1–16 fractions. The severity of RP was graded based on the CTCAE. RP progression was significantly correlated with the lung V5 and V10 indices. Nishimura et al. evaluated pulmonary and pleural reactions in 43 patients with inoperable NSCLC and no lymph node metastasis [17]. The patients were treated using 59.4–95.4 Gy (RBE) in 18 fractions. The lung V20 and V40 were determined to be significantly correlated with both the severity of pulmonary reactions and the incidence of pleural reactions. These two studies do not report specific values for these indices but recommend minimizing them to reduce the risk of severe complications.

## 3 Liver

Radiation‐induced liver damage (RILD) is a major complication after the radiotherapy of liver cancer or other upper abdominal cancers and can be life‐threatening. The treatment options are limited, and RILD can lead to fibrosis, cirrhosis, hepatitis, and liver failure [39, 40].

Hayashi et al. determined risk factors for RILD in 108 patients with hepatocellular carcinoma treated with 60 Gy (RBE) in 4 fractions [18]. RILD was defined as a worsening of two or more points in the Child–Pugh score within 12 months after CIRT. The study claimed that the normal liver volumes spared from less than 5–50 Gy (RBE) (VS5–50) were significant risk factors based on univariate analysis. However, only VS10 and VS30 had a *p*‐value less than 0.05. Cut‐off values were determined using ROC analysis. The significance level and multivariate analysis revealed that VS30 ≥ 739 cm^3^ is an independent risk factor for RILD. The one‐year cumulative incidences of RILD for VS30 < 739 cm^3^ and ≥ 739 cm^3^ were 39.6% and 9.2%, respectively.

## 4 Gastrointestinal Tract

Gastrointestinal (GI) tract toxicities are common after the treatment of thoracic, abdominal, and pelvic cancers if the GI organs are close to the target. Depending on the tumor type and severity of the toxicity, the patient's quality of life (QOL) may be significantly reduced [41]. Various papers have investigated correlations between DVH parameters and the incidence of acute and late GI complications, such as GI ulcers, intestine perforation, and rectal bleeding [19, 20, 21, 22, 23, 24, 25, 26, 27, 28].

### 4.1 Stomach

Predictive factors related to the incidence of upper GI ulcers were explored in 58 pancreatic cancer patients treated with a total dose of 55.2 Gy (RBE) delivered in 12 fractions [19]. GI ulcers were evaluated based on the CTCAE. DVH parameters were initially considered for the stomach, duodenum, and small intestine. However, ulcers developed only on the stomach, so only stomach parameters were used in the dosimetric analysis. Significant correlations were found between ulcer development and the stomach V10, V20, and V30. Using ROC analysis, the cut‐off values for V10, V20, and V30 were determined to be 102, 24, and 6 cm^3^, respectively. The actual incidences of gastric ulcer for each parameter were 51% versus 10% (stomach V10 ≥ 102 vs. < 102 cm^3^), 42% versus 9% (stomach V20 ≥ 24 vs. < 24 cm^3^), and 34% versus 4% (stomach V30 ≥ 6 vs. < 6 cm^3^). Moreover, it was investigated whether the constraint D2cm^3^ < 46 Gy (RBE) is a valid constraint for ulcer prevention. Based on the observations from a previous escalation study, it was indicated that the D2cm^3^ parameter of the GI tract should be kept below 46 Gy (RBE) to minimize ulcer occurrence [20]. During treatment planning, the D2cm^3^ index of GI tracts was kept under 46 Gy (RBE) [19]. No significant correlations were found between D2cm^3^ and ulcer occurrence. However, a low incidence of ulcers was observed compared with other photon and proton therapies; therefore, D2cm^3^ < 46 Gy (RBE) was concluded to be an appropriate constraint.

### 4.2 Lower GI Tract: Intestines, Rectum, and Sigmoid

Kato et al. conducted two clinical trials to evaluate the toxicity and efficacy of CIRT for locally advanced cervical cancer [21]. Forty‐four patients were treated with 16 fractions to the whole pelvis and an additional 8 fractions for local boost. The total dose was 52.8–72 Gy (RBE) for the first trial and 68.8–72.8 Gy (RBE) for the second trial. Patients in the first trial who received a dose greater than 60 Gy (RBE) to the GI tract due to overlap with the target developed grade ≥ 3 late GI morbidities. By strictly limiting the maximum dose (Dmax) under 60 Gy (RBE) in the second trial, no patient developed major GI toxicities. Although no significant differences were found between the development of GI toxicities and DVH parameters, these clinical observations support the use of GI tract (including the intestines, rectum and sigmoid colon) Dmax < 60 Gy (RBE) to prevent grade ≥ 3 late GI toxicities. This agrees with a different study that suggests adopting intestine Dmax < 60 Gy (RBE) as a constraint to avoid the development of intestine perforation; intestine perforation was observed in 4 out of 94 uterine cancer patients whose intestine received a dose that exceeded 60 Gy (RBE) [22].

### 4.3 Rectum and Sigmoid

Ishikawa et al. identified prognostic factors for late GI toxicities in 172 prostate cancer patients [23]. The total dose was 66 Gy (RBE) delivered in 20 fractions. GI complications were graded based on the Radiation Therapy Oncology Group/European Organization for Research and Treatment of Cancer Late Radiation Morbidity Scoring System (RTOG/EORTC). Univariate analysis revealed that V50 is significantly different between patients with and without rectal bleeding incidence. Multivariate analysis confirmed V50 > 13% to be an independent risk factor. The 5‐year cumulative rate of rectal bleeding was 12.3% for patients with V50 ≤ 13% and 22.2% for patients with V50 > 13%.

Normal tissue complication probability (NTCP) parameters were estimated for late rectal complications for 163 prostate cancer patients [24]. The patients were treated with a total dose of 57.6–72 Gy (RBE) in 16–20 fractions. GI complications were evaluated using the RTOG/EORTC. Dosimetric analysis showed that the doses at the leading edge of the NTCP curve were approximately 45 Gy (RBE) for grade ≥ 1 and 60 Gy (RBE) for grade ≥ 2 late rectal complications. Because the rectum behaves as a serial organ, the incident Dmax becomes important. Therefore, Dmax < 45 Gy (RBE) and Dmax < 60 Gy (RBE) were suggested as predictors for reducing grade ≥ 1 and grade ≥ 2 late rectal complications, respectively. The Dmax < 60 Gy (RBE) constraint agrees with the results of the two previous studies mentioned above [19, 20].

A study analyzed data from 259 prostate cancer patients treated with a total dose of 51.6 Gy (RBE) in 12 fractions [25]. Toxicities were classified using the CTCAE. ROC analysis revealed cut‐off values for the DVH parameters that had significant differences between the presence and absence of rectal 1 or 2 rectal bleeding: D6cm^3^ = 34.34 Gy (RBE), D2cm^3^ = 46.46 Gy (RBE), V10 = 9.85 cm^3^, V20 = 7.00 cm^3^, V30 = 6.91 cm^3^, and V40 = 4.26 cm^3^. Further analysis showed that D2cm^3^, V10, and V20 were significant risk factors for grade 2 rectal bleeding occurrence. The actual incidences of grade 2 rectal bleeding for each parameter were 7.7% versus 2.1% (D2cm^3^ ≥ 46.46 vs. < 46.46 Gy [RBE]), 5.9 versus 0.8% (V10 ≥ 9.85 vs. < 9.85 cm^3^), and 5.8% versus 0.8% (V20 ≥ 7.00 vs. < 7.00 cm^3^).

Okonogi et al. analyzed data from 132 patients with uterus carcinomas to determine predictive factors for late morbidities in the rectum [26]. The patients were treated with a total dose of 52.8–74.4 Gy (RBE) delivered in 20–24 fractions. Late toxicities were graded based on the RTOG/EORTC. There were no significant differences between late morbidity grades and DVH indices for the patients treated with 24 fractions. Univariate analysis showed that for the 20‐fraction group, the rectum D2cm^3^ and D5cm^3^ significantly correlated with the occurrence of grade ≥ 1 proctitis. Based on their significance, D2cm^3^ < 57.3 Gy (RBE) was selected as the constraint for preventing grade ≥ 1 proctitis.

Ohno et al. conducted the first phase I study about a new treatment regimen for advanced cervical cancer aiming to reduce severe late toxicities developing after a high‐dose CIRT boost: combined treatment of CIRT with three‐dimensional image‐guided brachytherapy (IGBT) [27]. Six patients were enrolled. CIRT consisted of whole‐pelvic irradiation with 36 Gy (RBE) in 12 fractions and a local boost with 19.2 Gy (RBE) in 4 fractions. After completion of CIRT, high dose‐rate brachytherapy was administered in 3 sessions. Acute and late toxicities were classified based on the CTCAE. The cumulative doses from the CIRT and IGBT were normalized to a biological equivalent dose of 2 Gy per fraction (EQD2) using a linear quadratic model with an alpha/beta ratio of 3 Gy for the OARs and 10 Gy for the tumor. The target accumulated dose to the rectum and sigmoid was D2cm^3^ = 67.2–71.3 Gy (RBE) EQD2. The dose‐limiting toxicities defined prior to the study were not observed, supporting the recommended dose for the rectosigmoid.

A recent study reported results for 15 patients with locally advanced cervical cancer that also received combined CIRT and IGBT using the same CIRT dosage as Ohno et al. [27, 28] Three sessions of IGBT were administered following CIRT. The target dose for the rectum and sigmoid was set to D2cm^3^ < 64.1 Gy. The dose was expressed in terms of the sum of the absolute dose from the CIRT and the EQD2 dose from the IGBT. Only one patient developed grade 2 sigmoid hemorrhage, who received D2cm^3^ = 65.6 Gy to the sigmoid. Although the evidence is weak, this suggests that restricting D2cm^3^ below 65.6 Gy may prevent the incidence of grade ≥ 2 sigmoid hemorrhage.

## 5 Bladder

Okonogi et al. also investigated predictive factors for late cystitis [26]. Radiation cystitis was graded based on the RTOG/EORTC. Similar to their results for proctitis, there were no significant differences between cystitis grades and DVH indices for the patients treated with 24 fractions. For the 20‐fraction group, significant differences were found in the bladder D5cm^3^ for the occurrence of grade ≥ 1 cystitis. Univariate analysis showed D5cm^3^ to be correlated with the development of cystitis, and multivariate analysis confirmed D5cm^3^ < 64.8 Gy (RBE) to be an independent prognostic factor for the occurrence of cystitis. Furthermore, it was observed that for doses to the bladder less than 20 Gy (RBE), patients without toxicities had higher doses than patients with toxicities. This implies that maintaining the dose to the bladder below 20 Gy (RBE) may prevent the development of cystitis.

## 6 Nerves

Radiation‐induced neuropathy is a rare chronic adverse effect, which could be progressive and irreversible, worsening the patient's QOL [42]. Imai et al. reported the results of CIRT for 188 patients with unresectable primary sacral chordomas [29]. A total dose of 64–73.6 Gy (RBE) was administered in 16 fractions. Adverse effects were graded using the CTCAE. Severe neuropathy was observed in six patients. In all six patients, the tumor was involved with the nerves, resulting in a high dose incident to a large volume of the nerves. Based on clinical observations, it was deduced that the dose incident to at least 10‐cm length (D10cm) of nerves and Dmax > 70 Gy (RBE) are risk factors for grade ≥ 3 neuropathy. Therefore, D10cm < 70 Gy (RBE) is suggested as a constraint.

A recent study identified DVH parameters that are associated with radiation‐induced lumbosacral plexopathy (RILSP) [30]. Twenty patients with pelvic recurrence of rectal cancer were treated with a total dose of 73.6 Gy (RBE) in 16 fractions. The patients had no RILSP prior to CIRT. RILSP was evaluated based on the CTCAE. ROC analysis revealed cut‐off values for grade ≥ 1 RILSP for the following DVH parameters: Dmax ≥ 74.44 Gy (RBE), D0.5cm^3^ ≥ 73.98 Gy (RBE), D1cm^3^ ≥ 73.88 Gy (RBE), D2cm^3^ ≥ 73.82 Gy (RBE), V20 ≥ 45.6%, V30 ≥ 44.4%, V40 ≥ 42.8%, V50% ≥ 33.2%, V60 ≥ 28.5%, and V70 ≥ 15.8%. The D2cm^3^ and V50–V70 indices were selected based on their area‐under‐the‐curve (AUC) values (D2cm^3^, AUC = 0.969; V50–70, AUC ≥ 0.953) and verified that they were significantly different between the RILSP‐positive and RILSP‐negative groups. The 2‐year cumulative incidences of RILSP for each parameter were 80.0% versus 0% (D2cm^3^ ≥ 73.82 vs. < 73.82 Gy [RBE]), 66.7% versus 0% (V50 ≥ 33.2% vs. < 33.2%), 72.7% versus 0% (V60 ≥ 28.5% vs. < 28.5%), and 66.7% versus 0% (V70 ≥ 15.8% vs. < 15.8%).

## 7 Skeletal System

Irradiation to the bones may lead to bone fracture and necrosis [31, 43]. In thoracic radiotherapy, radiation‐induced rib fracture (RIRF) is one of the possible late reactions that may occur after radiotherapy. In pelvic radiotherapy, femoral head necrosis is a serious complication. It can cause progressive pain, which can worsen due to weight‐bearing, and may lead to the loss of joint function [43]. Pelvic insufficiency fractures are also a possible adverse effect of pelvic radiotherapy that may decrease the QOL and increase mortality, particularly in elderly patients [44].

### 7.1 Ribs

One study assessed the relationship between DVH parameters and radiation‐induced rib fracture occurrence in lung cancer patients [31]. Data for 18 patients (57 ribs) treated with 52.8–60 Gy (RBE) in 4 fractions were analyzed. Significant differences were found in Dmax and V30–60 between fractured and non‐fractured ribs. ROC analysis was performed for the Dmax, D0.5–5 cm^3^, and V30–60 indices. The use of D1cm^3^ was suggested because it had the highest AUC (AUC = 0.78). The incidence rate of rib fracture was 53% for D1cm^3^ ≥ 38.2 Gy (RBE) and 4% for D1cm^3^ < 38.2 Gy (RBE).

### 7.2 Femoral Heads

Dosimetric prognostic factors for 29 patients with malignant pelvic bone sarcomas were investigated [32]. The total dose was 70.4–73.6 Gy (RBE) delivered in 16 fractions. Radiation‐induced pelvic fracture was defined as the progression of the bone displacement of the pelvis. DVH analysis showed that higher doses to the femoral head increased the frequency of necrosis. ROC analysis revealed that all DVH indices investigated (V10–65) had a high AUC (AUCs > 0.875). The use of V40 < 33% for predicting femoral head necrosis was suggested because V40 had the highest AUC value (AUC = 0.908). The cumulative incidence of femoral necrosis is significantly higher in patients with V40 ≥ 33% (about 80%) than in patients with V40 < 33% (0%). Moreover, a tolerance curve was created, which showed that the tolerable volume percentage of the femoral head decreased dramatically at doses higher than 30 Gy (RBE). The tolerance curve may be used during treatment planning to help determine beam directions.

### 7.3 Sacrum

Mori et al. examined risk factors for sacrum insufficiency fractures (SIFs) by analyzing data from 101 patients with uterine sarcoma [33]. The patients were treated with a total dose of 52.8–74.4 Gy (RBE) in 20–24 fractions. There were statistically significant differences in V10–30, D50%, D2cm^3^, and D5cm^3^ between patients with and without SIFs. By dividing the patients into groups based on the median value of each parameter, significant differences were observed in V10–40, D50%, and D2cm^3^. ROC analysis revealed that D50% and V20Gy had the highest AUC values: 0.755 and 0.753, respectively, indicating possible risk factors. Because the medium dose correlates with the SIF development, the study suggests the use of sacrum D50% < 19.9 Gy (RBE) as a constraint to prevent SIF occurrence, where 19.9 Gy (RBE) was the median value of D50%.

## 8 Skin

A common side effect of cancer radiotherapy, especially for cancers near the skin, is radiation‐induced skin reaction, which can affect the patient's QOL. Radiation‐induced skin reaction can manifest as either an acute or late reaction. DVH factors associated with acute skin reaction have already been reported in a previous review for head‐and‐neck cancer [12]. For this review, factors reported for non‐head‐and‐neck cancers are summarized.

Takakusagi et al. identified prognostic factors for grade ≥ 2 acute radiation dermatitis [34]. The study involved 22 malignant bone‐and‐soft‐tissue cancer patients treated with a total dose of 64–70.4 Gy (RBE) in 16 fractions. Skin severity was graded based on the CTCAE. There were significant differences in Dmax and the surface receiving 30–60 Gy (RBE) (S30–60) between patients with grade 1 and 2. S40 had the highest significance level among the dose–surface histogram indices. Cut‐off values for the occurrence of grade ≥ 2 acute radiation dermatitis were determined for Dmax and S40 using ROC analysis. These values were Dmax ≥ 52 Gy (RBE) and S40 ≥ 25 cm^2^.

Yanagi et al. investigated prognostic factors for the occurrence of both acute and late skin reactions, including ulceration [35]. Data from 35 patients (27 patients for late reactions) with unresectable bone and soft tissue sarcoma were evaluated. The patients were treated with 52.8–73.6 Gy (RBE) in 16 fractions. Acute reactions were graded based on the RTOG, and late reactions were based on the RTOG/EORTC. V64 > 100 mL and S60 > 20 cm^2^ were selected to be candidates for predicting late skin reactions. Multivariate analysis revealed only S60 > 20 cm^2^ to be an independent risk factor. The probability of grade 3 late skin reactions was 100% for S60 > 20 cm^2^ and 22.7% for S60 ≤ 20 cm^2^. The fact that only S60 was shown to be a factor indicates that even late skin reactions may be related to the irradiation of the superficial parts of the skin.

Table 1 lists all dose–volume constraints that were determined through previous studies for thoracic, abdominal, and pelvic cancers in CIRT.

**TABLE 1:** Dose–volume constraints that are suggested for clinical use based on prior research.

Organ	Parameter	Constraint	Tumor	Total dose	# of fractions	Clinical endpoint	Hard/soft constraint	Study reference
Bilateral lungs—GTV	V30	< 15%	Locally advanced NSCLC (including cases with lymph node metastasis)	68.0–76.0 Gy (RBE)	16	Grade ≥ 2 radiation pneumonitis	Soft	Hayashi 2017 [14]
	V5	≤ 11.0%	Solitary lung tumors (absence of lymph node metastasis)	50 Gy (RBE)	1	Grade ≥ 2 radiation pneumonitis	Soft	Ono 2021 [15]
	V10	≤ 9.4%					Soft	
	V15	≤ 7.8%					Soft	
	V20	≤ 6.8%					Soft	
	V25	≤ 4.5%					Soft	
	V30	≤ 3.5%					Soft	
	MLD	≤ 3.0 Gy (RBE)					Soft	
	V5	Minimize	NSCLC with interstitial lung disease (including cases with lymph node metastasis)	46.0–72.0 Gy (RBE)	1–16	Radiation pneumonitis grade progression (for patients with interstitial lung disease)	Soft	Nakajima 2017 [16]
	V10	Minimize					Soft	
	V20	Minimize	NSCLC (including cases with lymph node metastasis)	59.4–95.4 Gy (RBE)	18	Severity of pulmonary reactions and incidence of pleural reactions	Soft	Nishimura 2003 [17]
	V40	Minimize					Soft	
Liver	VS30	< 739 cm^3^	Hepatocellular carcinoma	60 Gy (RBE)	4	Radiation‐induced liver damage	Hard (Soft)^f^	Hayashi 2024 [18]
Stomach	D2cm^3^	< 46 Gy (RBE)	Pancreatic cancer	55.2 Gy (RBE)	12	Grade ≥ 1 gastric ulcer	Hard	Shinoto 2016 [19]
	V10	< 102 cm^3^					Soft	
	V20	< 24 cm^3^					Soft	
	V30	< 6 cm^3^					Hard	
Gastrointestinal tract	Dmax	< 60 Gy (RBE)	Locally advanced cervical cancer	52.8–72.8 Gy (RBE)	24	Grade ≥ 3 late GI complications	Hard (Hard)^d^	Kato 2006 [21]
	Dmax	< 60 Gy (RBE)	Advanced uterine cancer	52.8–72.8 Gy (RBE)	20–24	Intestine perforation	Hard (Hard)^d^	Matsushita 2006 [22]
Rectum	V50	< 13%	Prostate cancer	66.0 Gy (RBE)	20	Grade ≥ 1 late gastrointestinal toxicity	Soft	Ishikawa 2006 [23]
	Dmax	< 45 Gy (RBE)	Prostate cancer	57.6–72 Gy (RBE)	16–20	Grade ≥ 1 late rectum complications	Soft (Soft)^e^	Fukahori 2016 [24]
	Dmax	< 60 Gy (RBE)				Grade ≥ 2 late rectum complications	Soft (Hard)^e^	
	D2cm^3^	< 46.46 Gy (RBE)	Prostate cancer	51.6 Gy (RBE)	12	Grade ≥ 2 late rectum bleeding	Soft	Ono 2024 [25]
	V10	< 9.85 cm^3^						
	V20	< 7.00 cm^3^						
	D2cm^3^	< 57.3 Gy (RBE)	Uterus carcinoma	52.8–74.4 Gy (RBE)	20–24	Grade ≥ 1 late proctitis (20fr)	Soft	Okonogi 2018 [26]
Rectum and sigmoid	D2cm^3^	67.2–71.3 Gy (RBE) EQD2^a^	Cervical cancer	55.2 Gy (RBE) + IGBT^b^	16 + 3	Protocol constraint	Soft	Ohno 2018 [27]
	D2cm^3^	< 65.6 Gy (RBE) EQD2^c^	Cervical cancer	55.2 Gy (RBE) + 16.5Gy (IGBT)	16 + 3	Grade ≥ 2 late sigmoid hemorrhage	Soft	Tsuchida 2024 [28]
Bladder	D5cm^3^	< 64.8 Gy (RBE)	Uterus carcinoma	52.8–74.4 Gy (RBE)	20–24	Grade ≥ 1 late cystitis (20fr)	Soft	Okonogi 2018 [26]
Nerves	D10cm	< 70 Gy (RBE)	Unresectable primary sacral chordoma	64–73.6 Gy (RBE)	16	Grade ≥ 3 neuropathy	Soft	Imai 2016 [29]
	D2cm^3^	< 73.82 Gy (RBE)	Pelvic recurrence of rectal cancer	73.6 Gy (RBE)	16	Grade ≥ 1 radiation‐induced lumbosacral plexopathy	Soft	Kumazawa 2025 [30]
	V50	< 33.2%						
	V60	< 28.5%						
	V70	< 15.8%						
Rib	D1cm^3^	< 38.2 Gy (RBE)	Lung cancer	52.8 or 60.0 Gy (RBE)	4	Radiation‐induced rib fracture	Soft (Soft)^f^	Abe 2016 [30]
Femoral head	V40	< 33%	Malignant pelvic bone sarcoma	70.4–73.6 Gy (RBE)	16	Femoral head necrosis	Soft	Takenaka 2020 [31]
Sacrum	D50%	< 19.9 Gy (RBE)	Uterine sarcoma	52.8–74.4 Gy (RBE)	20–24	Sacrum insufficiency fracture	Soft (Soft)^e^	Mori 2022 [32]
Skin	Dmax	< 52 Gy (RBE)	Malignant bone‐and‐soft‐tissue cancer	64.0–70.4 Gy (RBE)	16	Grade ≥ 2 acute radiation dermatitis	Hard	Takakusagi 2017 [33]
	S40	< 25 cm^2^					Soft	
	S60	< 20 cm^2^	Unresectable bone‐and‐soft‐tissue sarcoma	52.8–73.6 Gy (RBE)	16	Grade ≥ 3 late skin reaction	Hard	Yanagi 2010 [34]

Abbreviations: Dmax, maximum dose; Dxx, dose incident to xx cm/cm^3^/% length/volume of organ at risk; EQD2, biological equivalent dose of 2 Gy per fraction; fr, fractions; GTV, gross tumor volume; IGBT, image‐guided brachytherapy; MLD, mean lung dose; NSCLC, non‐small cell lung cancer; Sxx, surface area receiving a dose of xx Gy (RBE); VSxx, normal liver volume spared from less than xx Gy (RBE); Vxx, volume of organ at risk receiving a dose of xx Gy (RBE).

^a^ Normalized to EQD2.

^b^ The IGBT dose was varied to meet the recommended dose for the rectosigmoid.

^c^ Sum of the absolute dose of carbon ion radiotherapy and the EQD2 from high dose‐rate brachytherapy.

^d^ Corresponding to abdominal cancers, including bone‐and‐soft tissue cancer.

^e^ Corresponding to pelvic cancers, including bone‐and‐soft‐tissue cancer.

^f^ Corresponding to thoracic cancers, including bone‐and‐soft‐tissue cancer.

## 9 Discussion

All dose–volume constraints reported to be associated with adverse effects in thoracic, abdominal, and pelvic CIRT through previous research based on the Japanese RBE models are summarized. This review may be a step towards formally establishing constraints in CIRT. The establishment of constraints is important in optimizing treatment plans for each patient by ensuring adverse effects are minimized. Furthermore, establishing constraints would enable multicenter studies and comparisons between different modalities, and facilitate adaptive radiotherapy in CIRT.

It has been previously discussed that the notion of hard and soft constraints should be considered not only in advanced treatment techniques, such as intensity‐modulated radiotherapy, volumetric‐modulated arc therapy, and intensity‐modulated proton therapy, but also in CIRT [12]. Table 1 classifies the constraints into hard and soft. The classification was first carried out based on the type of tumor listed in Table 1 for which the constraints were determined. Classifications in parentheses correspond to different tumor types. The constraints were divided based on various factors, including the severity of the complication, whether a complication can be treated, and how easy it is to meet the constraint based on the prescription dose and whether priority should be given to the target coverage. For example, the Dmax constraints for the GI tract were categorized as hard regardless of the type of cancer, because complications such as intestine perforation are severe. Similarly, the skin Dmax and S60 constraints were set to hard because not meeting these constraints may cause severe ulcers and abscesses. On the other hand, constraints for the rib, femoral head, and sacrum were set to soft. Although it is preferable to avoid irradiating these OARs, target coverage should be prioritized since complications are not fatal, especially for bone‐and‐soft tissue cancers, which are radioresistant. The table can be used during treatment planning to facilitate decisions on which constraints to prioritize or whether more weight should be given to tumor coverage or OAR sparing if necessary.

Several strategies can be adopted to ensure that radiation to the OARs is minimized. One such strategy is the shrinking field technique. Shrinking the clinical target volume twice according to tumor shrinkage has been shown to lead to reduced toxicities in cervical cancer treatment [45]. Additionally, the choice of irradiation techniques may also result in reduced doses to the OARs. This has already been discussed in a previous article [12]. However, for thoracic, abdominal, and pelvic cancers, the uncertainties arising from the target motion due to respiration and the anatomical variations due to OAR motion and changes in the OAR volume need to also be considered. Respiratory gating is generally being applied for thoracoabdominal cancers to mitigate respiratory effects [16, 17, 21, 31]. Furthermore, in case the tumor is located close to the GI tract, a displacement device, called a spacer, can be placed to physically separate the tumor and the GI tract and consequently lower the dose to the GI tract [46, 47]. There are different types of spacers, such as in vivo tissue, artificial, and biodegradable. Saline injection to the bladder and a vaginal immobilization device can also be used to separate the tumor site from the GI tract and minimize intrafractional motion [26, 27]. Moreover, since a limited number of beams is used in CIRT, beam angle arrangement becomes important in choosing angles that avoid OARs and mitigate interfractional and intrafractional variations [48, 49]. Another strategy would be to use DVH and NTCP models derived from CIRT‐treated patient data during plan optimization. By aiming to conform the organ DVH/NTCP of a plan to the limits specified by a DVH/NTCP model would potentially lead to fewer complications [50].

As mentioned in the Introduction, Japan and Germany have the longest clinical experience in CIRT, indicating that CIRT facilities can benefit from their long‐term data. However, the differences in the RBE models render impossible the direct application of prescription doses and constraints that are based on a different RBE model than the model used at one's own institution. Studies have shown that Japanese RBE‐weighted doses can be lower, similar to, or higher than LEM RBE‐weighted doses depending on the fraction dose [51, 52]. Therefore, LEM‐based institutions that wish to use Japanese data will have to apply appropriate conversion factors for biological dose conversion [51, 52, 53]. Previous studies have attempted to translate prescription doses and constraints between Japanese models and LEM [10, 54, 55, 56, 57, 58, 59]. Fossati et al. defined LEM‐based prescription doses for head‐and‐neck cancer by developing a simple calculation method to translate Japanese clinical experience data and minimize the physical dose differences between the two models [60]. Gora et al. recently reported various scenarios where translations between the models diverge and highlighted the benefit of utilizing both Japanese and LEM‐based constraints during treatment planning [52]. Optimal planning strategies for minimizing such differences were also reported, contributing to the application of published conversion methods for escalating dose constraints for specific OARs. These conversion methods depend on various parameters, such as the spread‐out Bragg peak width and depth, fractionation, tissue characteristics, and endpoint [51, 52, 53]. This implies that prior to using a conversion method, all specific parameters used to develop the respective method must be considered. Moreover, the tumor sites and OARs investigated are limited. Future research should focus on determining conversion factors for all tumors treated with CIRT and their respective OARs. This would enable harmonization of dose–volume constraints between Japanese and LEM‐based institutions, supporting collaborative research initiatives and advancing the global implementation of CIRT.

This review focuses on the dose–volume constraints for CIRT that were determined through prior research. However, CIRT institutions typically employ a more comprehensive set of constraints for a broader range of OARs than those presented here. Constraints not included in this review are generally determined based on clinical experience, with institutions referencing DVH parameters from past cases where treatment was safely delivered [61, 62, 63, 64]. Examples of such constraints that have been reported are shown in Table S1. While these experience‐based constraints have demonstrated empirical safety, they leave uncertainties regarding dose escalation limits and toxicity thresholds since they were not derived from formal dose escalation trials. Institutions may also adopt constraints from organs with similar radiobiological properties for those that have not been investigated yet. Consequently, for OARs not covered in this study and pending the establishment of a formal consensus, clinicians can determine constraints by combining their institution's accumulated clinical experience, the radiobiological properties of the OAR in question, and extrapolation from similar and well‐studied OARs. Moreover, clinicians should adjust the constraints presented in this study when applying them to different fractionation regimens. Previous studies have used the LQ model to convert constraints between fractionation regimens, particularly when comparing Japanese models with the LEM [65, 66]. The converted constraints, though, were not validated in clinical practice. A recent paper introduced a new metric, called fractionation‐specific biological equivalent dose, for adjusting dose constraints across various fractionation regimens in XRT [67]. This metric, which was validated against XRT clinical data, could be adjusted and validated for CIRT. Nevertheless, biological models remain speculative and lack sufficient clinical validation for CIRT. Caution is necessary when using these models, and any adjustments to constraints should be supported by clinical experience and biological experiments. On a positive note, Japan's standardized fractionation protocols limit the variety of regimens, making the reviewed data largely representative of the current CIRT practice. Although the reviewed data are not exhaustive, this review serves as a foundation for future refinement and expansion.

Most studies that report on the outcomes of CIRT mainly focus on reporting tumor control, survival rates, and toxicity incidences. Unfortunately, the number of papers that report the association between DVH parameters and toxicity incidence is few. The majority of such studies were conducted using a small sample of patients with various fractionation schemes. Furthermore, some papers used poor‐quality methodologies to determine constraints, such as clinical observations and the significance level (*p*‐value). These suggest that the constraints determined may not be reliable either due to the small sample size and non‐uniform treatment schemes, and/or the method used to select significant risk factors. Table S2 lists all constraints found to be associated with toxicity development before determining risk factors. This table may be used as a supplementary reference guide during planning. The reliability of the dose–volume constraints has to be verified through studies with a sufficiently large number of patients. Such large‐scale studies should also focus on investigating patients with varying lung and liver function. The constraints between patients with good and bad lung/liver function are expected to differ. It has already been suggested that lung V5 and V10 should be minimized in patients with interstitial lung disease to prevent RP grade progression; however, no specific values were stated [16]. Moreover, studies on pediatric cancer CIRT are sparse and do not report DVH‐based risk factors [68, 69, 70]. Although randomized control trials cannot be conducted for pediatric patients due to ethical concerns, retrospective studies should be performed to evaluate the use of the current constraints. Also, constraints for the esophagus, trachea, bronchus, stomach, kidneys, and spinal cord have yet to be determined based on research. Further research is necessary to obtain a thorough list of the dose–volume constraints for thoracic, abdominal, and pelvic CIRT.

## 10 Conclusions

Developing a general consensus for dose–volume constraints is important for improving the safety and efficacy of CIRT. However, research is still insufficient, and the formulation of guidelines will probably take a long time to be finalized. This review constitutes one of the first steps in formally establishing constraints for CIRT. We presented all constraints determined for thoracic, abdominal, and pelvic CIRT that are based on the Japanese RBE models. The list provided can be used as a guide during treatment planning to optimize plans and ensure complications are minimized.

## Author Contributions

**Maria Varnava:** conceptualization (equal); investigation (lead); methodology (lead); resources (equal); Visualization (lead); writing – original draft preparation (lead); writing – review and editing (equal). **Mutsumi Tashiro:** resources (supporting); writing – review and editing (equal). **Masahiko Okamoto:** resources (supporting); visualization (supporting); writing – review and editing (equal). **Ken Ando:** resources (supporting); visualization (supporting); writing – review and editing (equal). **Nobutero Kubo:** resources (supporting); visualization (supporting); writing – review and editing (equal). **Hidemasa Kawamura:** resources (supporting); visualization (supporting); writing – review and editing (equal). **Masahiro Onishi:** writing – review and editing (equal). **Kei Shibuya:** writing – review and editing (equal). **Takuya Kumazawa:** writing – review and editing (equal). **Takeru Ohtaka:** writing – review and editing (equal). **Tatsuya Ohno:** conceptualization (lead); investigation (equal); resources (equal); writing – review and editing (equal).

## Ethics Statement

The authors have nothing to report.

## Conflicts of Interest

The authors declare no conflicts of interest.

## Supporting information


Tables S1 and S2.

## Data Availability

The authors have nothing to report.
