# Supplementary material for: Dose–Volume Constraints for Thoracic, Abdominal, and Pelvic Carbon Ion Radiotherapy: A Literature Review
Source: Cancer Med. 2025 Mar 28;14(7):e70840. doi: 10.1002/cam4.70840 (PMC11953175; doi:10.1002/cam4.70840)
Supplement: Supplementary file 1 — Tables S1 and S2. [file CAM4-14-e70840-s001.docx]

Supplementary Table S1. Dose–volume constraints for organs-at-risk that are being used in clinical practice without published evidence.

| **Organ** | **Parameter** | **Constraint** | **Tumor** | **Total dose** | **# of fractions** | **Study reference** |
| --- | --- | --- | --- | --- | --- | --- |
| Spinal cord | Dmax | <30 Gy (RBE) | Locally advanced NSCLC (including cases with lymph node metastasis) | 64.0–76.0 Gy (RBE) | 16 | Anzai 2020^61^ |
|  | Dmax | <30 Gy (RBE) | Isolated lymph node metastasis | 48.0–52.8 Gy (RBE) | 12 | Shirai 2019^62^ |
|  | Dmax | <30 Gy (RBE) | Soft tissue sarcoma | 67.2–70.4 Gy (RBE) | 16 | Takakusagi 2022^63^ |
|  | Dmax | ≤25 Gy (RBE) | Esophageal carcinoma | 28.8–36.8 Gy (RBE) | 8 | Tsujii 2014^64^ |
|  | Dmax | ≤30 Gy (RBE) | Locally advanced pancreatic cancer | 55.2 Gy (RBE) | 12 |  |
|  |  |  | Resectable pancreatic cancer | 35.2 Gy (RBE) | 8 |  |
| Esophagus | Dmax | <50 Gy (RBE) | Locally advanced NSCLC (including cases with lymph node metastasis) | 64.0–76.0 Gy (RBE) | 16 | Anzai 2020^61^ |
|  | V50 | Minimize | Isolated lymph node metastasis | 48.0–52.8 Gy (RBE) | 12 | Shirai 2019^62^ |
| Bronchus | Dmax | <60 Gy (RBE) | Locally advanced NSCLC (including cases with lymph node metastasis) | 64.0–76.0 Gy (RBE) | 16 | Anzai 2020^61^ |
|  | V50 | Minimize | Isolated lymph node metastasis | 48.0–52.8 Gy (RBE) | 12 | Shirai 2019^62^ |
| Trachea | V50 | Minimize | Isolated lymph node metastasis | 48.0–52.8 Gy (RBE) | 12 | Shirai 2019^62^ |
| Renal parenchyma | V50 | ≤15 Gy (RBE) | Locally advanced pancreatic cancer | 55.2 Gy (RBE) | 12 | Tsujii 2014^64^ |
|  |  |  | Resectable pancreatic cancer | 35.2 Gy (RBE) | 8 |  |

Abbreviations: Dmax, maximum dose; V50, volume of organ at risk receiving a dose of 50 Gy (RBE).

Supplementary Table S2. All dose–volume constraints that were initially considered to be significant predictors of adverse effects prior to using a selection method.

| **Organ** | **Parameter** | **Constraint** | **Tumor** | **Total dose** | **# of fractions** | **Clinical endpoint** | **Selection method** | **Study reference** |
| --- | --- | --- | --- | --- | --- | --- | --- | --- |
| Bilateral lungs - GTV | Dmean | <12.5 Gy (RBE) | Locally advanced NSCLC (including cases with lymph node metastasis) | 68.0–76.0 Gy (RBE) | 16 | Grade ≥ 2 radiation pneumonitis | Multivariate analysis | Hayashi 2017^14^ |
|  | V5 | <28.8% |  |  |  |  |  |  |
|  | V10 | <29.9% |  |  |  |  |  |  |
|  | V20 | <20.1% |  |  |  |  |  |  |
|  | **V30** | **<15%**† |  |  |  |  |  |  |
|  |  |  |  |  |  |  |  |  |
|  | **V5** | **≤11.0%**† | Solitary lung tumors  (absence of lumph node metastasis) | 50 Gy (RBE) | 1 | Grade ≥ 2 radiation pneumonitis | Univariate analysis | Ono 2021^15^ |
|  | **V10** | **≤9.4%**† |  |  |  |  |  |  |
|  | **V15** | **≤7.8%**† |  |  |  |  |  |  |
|  | **V20** | **≤6.8%**† |  |  |  |  |  |  |
|  | **V25** | **≤4.5%**† |  |  |  |  |  |  |
|  | **V30** | **≤3.5%**† |  |  |  |  |  |  |
|  | **MLD** | **≤3.0 Gy (RBE)**† |  |  |  |  |  |  |
|  |  |  |  |  |  |  |  |  |
|  | **V5** | **Minimize**† | NSCLC with interstitial lung disease (including cases with lymph node metastasis) | 46.0–72.0 Gy (RBE) | 1–16 | Radiation pneumonitis grade progression (for patients with interstitial lung disease) | Significance (p-value) | Nakajima 2017^16^ |
|  | **V10** | **Minimize**† |  |  |  |  |  |  |
|  |  |  |  |  |  |  |  |  |
|  | **V20** | **Minimize**† | NSCLC (including cases with lymph node metastasis) | 59.4–95.4 Gy (RBE) | 18 | Severity of pulmonary reactions and incidence of pleural reactions |  | Nishimura 2003^17^ |
|  | **V40** | **Minimize**† |  |  |  |  |  |  |
| Liver | VS5 | <637 cm^3^ | Hepatocellular carcinoma | 60 Gy (RBE) | 4 | Radiation-induced liver damage | Significance level & Multivariate analysis | Hayashi 2024^18^ |
|  | VS10 | <506 cm^3^ |  |  |  |  |  |  |
|  | VS15 | <697 cm^3^ |  |  |  |  |  |  |
|  | VS20 | <714 cm^3^ |  |  |  |  |  |  |
|  | **VS30** | **<739 cm^3^**† |  |  |  |  |  |  |
|  | VS40 | <937 cm^3^ |  |  |  |  |  |  |
|  | VS50 | <968 cm^3^ |  |  |  |  |  |  |
| Stomach | **D2cm^3^** | **<46 Gy (RBE)**† | Pancreatic cancer | 55.2 Gy (RBE) | 12 | Grade ≥ 1 gastric ulcer | Clinical observations | Shinoto 2016^19^ |
|  | **V10** | **<102 cm^3^**† |  |  |  |  | Significance (correlation & *p*-value) |  |
|  | **V20** | **<24 cm^3^**† |  |  |  |  |  |  |
|  | **V30** | **<6 cm^3^**† |  |  |  |  |  |  |
| Gastrointestinal tract | **Dmax** | **<60 Gy (RBE)**† | Locally advanced cervical cancer | 52.8–72.8 Gy (RBE) | 24 | Grade ≥ 3 late GI complications | Clinical observations | Kato 2006^21^ |
|  |  |  |  |  |  |  |  |  |
|  | **Dmax** | **<60 Gy (RBE)**† | Advanced uterine cancer | 52.8–72.8 Gy (RBE) | 20–24 | Intestine perforation | Clinical observations | Matsushita 2006^22^ |
| Rectum | **V50** | **<13%**† | Prostate cancer | 66.0 Gy (RBE) | 20 | Grade ≥ 1 late gastrointestinal toxicity | Multivariate analysis | Ishikawa 2006^23^ |
|  |  |  |  |  |  |  |  |  |
|  | **Dmax** | **<45 Gy (RBE)**† | Prostate cancer | 52.8–74.4 Gy (RBE) | 20–24 | Grade ≥ 1 late rectum complications | NTCP analysis | Fukahori 2016^24^ |
|  | **Dmax** | **<60 Gy (RBE)**† |  |  |  | Grade ≥ 2 late rectum complications |  |  |
|  |  |  |  |  |  |  |  |  |
|  | **D2cm^3^** | **<46.46 Gy (RBE)**† | Prostate cancer | 51.6 Gy (RBE) | 12 | Grade ≥ 2 late rectum bleeding | Correlation analysis between AUC values and complication | Ono 2024^25^ |
|  | D6cm^3^ | <34.34 Gy (RBE) |  |  |  |  |  |  |
|  | **V10** | **<9.85 cm^3^**† |  |  |  |  |  |  |
|  | **V20** | **<7.00 cm^3^**† |  |  |  |  |  |  |
|  | V30 | <6.91 cm^3^ |  |  |  |  |  |  |
|  | V40 | <4.26 cm^3^ |  |  |  |  |  |  |
|  |  |  |  |  |  |  |  |  |
|  | **D2cm^3^** | **<57.3 Gy (RBE)**† | Uterus carcinoma | 52.8–74.4 Gy (RBE) | 20–24 | Grade ≥ 1 late proctitis (20fr) | Multivariate analysis | Okonogi 2018^26^ |
|  | D5cm^3^ | <52.1 Gy (RBE) |  |  |  | Grade ≥ 1 late proctitis (20fr) |  |  |
| Rectum and sigmoid | **D2cm^3^** | **67.2-71.3 Gy (RBE) EQD2**†^a^ | Cervical cancer | 55.2 Gy (RBE) + IGBT^b^ | 16 + 3 | Protocol constraint | - | Ohno 2018^27^ |
|  |  |  |  |  |  |  |  |  |
|  | **D2cm^3^** | **＜65.6 Gy (RBE) EQD2**†^c^ | Cervical cancer | 55.2 Gy (RBE) + 16.5Gy (IGBT) | 16 + 3 | Grade ≥ 2 late sigmoid hemorrhage | Clinical observations | Tshichida 2024^28^ |
| Bladder | **D5cm^3^** | **<64.8 Gy (RBE)**† | Uterus carcinoma | 52.8–74.4 Gy (RBE) | 20–24 | Grade ≥ 1 late cystitis (20fr) | Multivariate analysis | Okonogi 2018^26^ |
| Nerves | **D10cm** | **<70 Gy (RBE)**† | Unresectable primary sacral chordoma | 64–73.6 Gy (RBE) | 16 | Grade ≥ 3 neuropathy | Clinical observations | Imai 2016^29^ |
|  |  |  |  |  |  |  |  |  |
|  | Dmax | <74.44 Gy (RBE) | Pelvic recurrence of rectal cancer | 73.6 Gy (RBE) | 16 | Grade ≥ 1 radiation-induced lumbosacral plexopathy | AUC value | Kumazawa 2025^30^ |
|  | Dmean | Not specified |  |  |  |  |  |  |
|  | D0.5cm^3^ | <73.98 Gy (RBE) |  |  |  |  |  |  |
|  | D1cm^3^ | <73.88 Gy (RBE) |  |  |  |  |  |  |
|  | **D2cm^3^** | **<73.82 Gy (RBE)**† |  |  |  |  |  |  |
|  | V20 | <45.6% |  |  |  |  |  |  |
|  | V30 | <44.4% |  |  |  |  |  |  |
|  | V40 | <42.8% |  |  |  |  |  |  |
|  | **V50** | **<33.2%**† |  |  |  |  |  |  |
|  | **V60** | **<28.5%**† |  |  |  |  |  |  |
|  | **V70** | **<15.8%**† |  |  |  |  |  |  |
| Rib | **D1cm^3^** | **<38.2 Gy (RBE)**† | Lung cancer | 52.8 or 60.0 Gy (RBE) | 4 | Radiation-induced rib fracture | AUC value | Abe 2016^30^ |
|  | Dmax | <49.7 Gy (RBE) |  |  |  |  |  |  |
|  | V30 | <4.7 cm^3^ |  |  |  |  |  |  |
|  | V40 | <0.9 cm^3^ |  |  |  |  |  |  |
|  | V50 | Not reported |  |  |  |  |  |  |
|  | V60 | <0.1 cm^3^ |  |  |  |  |  |  |
| Femoral head | V10 | <100% | Malignant pelvic bone sarcomas | 70.4–73.6 Gy (RBE) | 16 | Femoral head necrosis | AUC value & tolerance curve | Takenaka 2020^31^ |
|  | V15 | <100% |  |  |  |  |  |  |
|  | V20 | <100% |  |  |  |  |  |  |
|  | V25 | <96% |  |  |  |  |  |  |
|  | V30 | <40% |  |  |  |  |  |  |
|  | V35 | <36% |  |  |  |  |  |  |
|  | **V40** | **<33%**† |  |  |  |  |  |  |
|  | V45 | <29% |  |  |  |  |  |  |
|  | V50 | <24% |  |  |  |  |  |  |
|  | V55 | <20% |  |  |  |  |  |  |
|  | V60 | <16% |  |  |  |  |  |  |
|  | V65 | <11% |  |  |  |  |  |  |
| Sacrum | V10 | <159.1 cm^3^ | Uterine sarcoma | 52.8–74.4 Gy (RBE) | 20–24 | Sacrum insufficiency fracture | AUC value & clinical observations | Mori 2022^32^ |
|  | V20 | <92.1 cm^3^ |  |  |  |  |  |  |
|  | V30 | <19.4 cm^3^ |  |  |  |  |  |  |
|  | **D50%** | **<19.9 Gy (RBE)**† |  |  |  |  |  |  |
|  | D2cm^3^ | <36.8 Gy (RBE) |  |  |  |  |  |  |
|  | D5cm^3^ | <39.0 Gy (RBE) |  |  |  |  |  |  |
| Skin | **Dmax** | **<52 Gy (RBE)**† | Malignant bone-and-soft-tissue cancer | 64.0–70.4 Gy (RBE) | 16 | Grade ≥ 2 acute radiation dermatitis | Univariate analysis &  Significance (p-value) | Takakusagi 2017^33^ |
|  | S30 | Not reported |  |  |  |  |  |  |
|  | **S40** | **<25 cm^2^**† |  |  |  |  |  |  |
|  | S50 | Not reported |  |  |  |  |  |  |
|  | S60 | Not reported |  |  |  |  |  |  |
|  |  |  |  |  |  |  |  |  |
|  | V64 | <100 cm^3^ | Unresectable bone-and-soft-tissue sarcoma | 52.8–73.6 Gy (RBE) | 16 | Grade ≥ 3 late skin reaction | Multivariate analysis | Yanagi 2010^34^ |
|  | **S60** | **<20 cm^2^**† |  |  |  |  |  |  |

Abbreviations: AUC, area under the curve; EQD2, biological equivalent dose of 2 Gy per fraction; fr, fractions; GTV, gross tumor volume; Dmax, maximum dose; Dmean, mean dose; Dxx, dose incident to xx cm/cm^3^/% volume of organ at risk; MLD, mean lung dose; NSCLC, non-small cell lung cancer; Sxx, surface area receiving a dose of xx Gy (RBE);, VSxx, normal liver volume spared from less than xx Gy (RBE), Vxx, volume of organ at risk receiving a dose of xx Gy (RBE).

All parameters listed were determined to be significantly related to respective toxicities using statistical methods. The selection method refers to the method used to determine which parameters are significant risk factors.

†Parameters highlighted in bold correspond to the parameters determined by the selection method. These constraints are suggested to be used as constraints by previous research.

^a^normalized to EQD2

^b^the IGBT dose was varied to meet the recommended dose for the rectosigmoid

^c^sum of the absolute dose of carbon ion radiotherapy and the EQD2 from high dose-rate brachytherapy
